# Quantifying Soft‐Surgery in Cochlear Implantation: Multimodal Data From 30 International Specialists

**DOI:** 10.1002/lary.70550

**Published:** 2026-04-09

**Authors:** Philipp Aebischer, Georgios Mantokoudis, Marco Caversaccio, Stefan Weder

**Affiliations:** ^1^ Hearing Research Laboratory, ARTORG Center for Biomedical Engineering Research University of Bern Bern Switzerland; ^2^ Department for Otolaryngology, Head and Neck Surgery Inselspital University Hospital Bern Bern Switzerland

**Keywords:** cochlear implant, intracochlear pressure, multi‐sensor temporal bone model, soft‐surgery technique

## Abstract

**Objective:**

To quantify how surgical behavior influences intracochlear mechanical stress during cochlear implantation using objective multi‐sensor data from a large cohort of specialist cochlear implant (CI) surgeons.

**Methods:**

Thirty internationally recognized surgeons each performed bilateral insertions in mechanically representative artificial temporal bone models. Insertion force, pressure, and electrode kinematics were captured, and six trauma‐related metrics were combined into a composite soft‐surgery score. Associations with surgical experience, self‐assessment, and handling behaviors were analysed using nonparametric statistics.

**Results:**

Marked variability was observed across all metrics. Surgeons with < 50 lifetime insertions performed significantly worse than experienced colleagues. Self‐assessment showed no correlation with objective outcomes. Excessively slow insertions and frequent electrode regrasping were associated with higher pressure exposure, greater force variation, and increased intracochlear implant motion. Post‐insertion handling contributed substantially to intracochlear stress.

**Conclusion:**

Surgical behavior decisively influences intracochlear mechanical stress in CI. Steady, uninterrupted advancement at slow to moderate speed, avoidance of array regrasping, and careful post‐insertion handling reduce mechanical load. Because self‐assessment proved unreliable even among specialist CI surgeons, structured training with quantitative feedback and intraoperative monitoring is essential to refine soft‐surgery technique, preserve residual hearing, and optimize hearing outcomes.

**Level of Evidence:**

N/A.

## Graphical abstract

Objective data from 30 specialist cochlear implant surgeons showed that surgical handling strongly influences intracochlear mechanical stress during implantation. Surgeons with fewer than 50 lifetime insertions performed significantly worse than more experienced colleagues, while self‐assessment did not reflect objective performance in any group. Steady slow‐to‐moderate insertions, minimal regrasping, and careful post‐insertion handling reduced mechanical load. These results support structured training with quantitative feedback to improve soft‐surgery technique and hearing preservation.

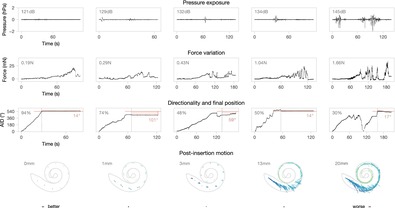

## 1 Introduction

Cochlear implants (CIs) are highly effective for restoring hearing in individuals with severe to profound hearing loss. Their use, however, remains limited in patients with substantial residual hearing because implantation often deteriorates the remaining natural hearing. This barrier constrains adoption and excludes many candidates who might otherwise benefit.

To address this challenge, the concept of *soft surgery* was introduced by Lehnhardt in 1993 and later refined by Laszig, Friedland, and Lenarz [1, 2, 3, 4]. These recommendations emphasize gentle cochlear access, controlled electrode advancement, minimal force, and generous lubrication. Yet, because cochlear microtrauma is invisible on clinical imaging, direct quantification of intracochlear stress in vivo remains difficult, and translation from principle to practice still relies largely on expert opinion rather than empirical evidence.

Where direct patient measurement is limited, complementary evidence comes from cadaveric temporal bone studies and mechanically representative temporal bone models. These systems show that different elements of surgical technique generate distinct mechanical stresses. On a microscopic scale, hand tremor and micromovements are transmitted through the electrode array, displacing perilymph and producing intracochlear pressure transients that may damage hair‐cell stereocilia and cause permanent sensorineural hearing loss [5]. On a macroscopic scale, friction and the array's bending stiffness during insertion produce forces that rise exponentially with depth and can exceed tissue‐damage thresholds [6, 7].

These interacting forces also shape the trajectory of the array at greater depths. As friction increases in the second half of insertion, longitudinal motion may convert into transverse bulging or buckling within the basal scala tympani [8], raising the risk of contact with the basilar membrane, spiral lamina, or modiolus. Such deformation generates compressive stress that can tear the basilar membrane or fracture bony structures [7, 9]. Importantly, implantation does not end with placement of the array: releasing the forceps and routing the lead through the mastoidectomy can provoke millimeter‐scale electrode shifts, producing mechanical and pressure transients comparable to those during insertion [10]. Electrophysiology confirms that these late‐phase events acutely stress cochlear function [11].

Beyond immediate mechanical events, tissue responses unfold over time. Surgical trauma initiates inflammation, fibrosis, and intracochlear neo‐ossification [12]. Even without overt injury, compression of the spiral ligament can disturb ionic homeostasis [13]. These chronic changes degrade residual acoustic hearing, reduce the efficacy of electrical stimulation, and diminish postoperative performance. In addition, mismatch between intended and achieved insertion depth limits cochlear coverage and tonotopic mapping [14].

Despite this body of evidence, the contribution of individual surgical behavior to mechanical stress remains unclear, as does the extent to which such effects are perceptible to surgeons or modifiable through experience.

To address these gaps, we conducted a multinational in vitro trial enrolling a large cohort of recognized CI specialists. Using a mechanically representative, multi‐sensor temporal bone model, we combined pressure and force measurements with kinematic data to quantify mechanical stress throughout the implantation workflow. We then integrated six phase‐specific metrics into a composite soft‐surgery score that enables objective comparison across procedures and surgeons.

We tested three hypotheses: (1) surgical experience correlates with higher soft‐surgery scores; (2) surgeons can reliably self‐assess their performance; and (3) specific, actionable behaviors explain inter‐surgeon variability. The first two assess whether anecdotal guidelines are supported by objective measurement, whereas the third examines how insights from the multi‐sensor data can guide behavioral changes that improve outcomes. By linking surgical behavior to quantitative stress metrics, this study provides an evidence‐based foundation for refining soft‐surgery principles.

## 2 Materials and Methods

We conducted a multinational, in vitro study in which 30 internationally recognized specialist CI surgeons performed bilateral CI insertions in artificial temporal bone models. All surgeons were invited contributors in their role as CI surgeons to the XXI Hearing and Structure Preservation Workshop (Bern, Switzerland), and were required to have clinical experience performing cochlear implantation. Participants provided informed consent for analysis of anonymized data. For each surgeon, we recorded years of CI practice, cumulative clinical insertions (binned as 0–50, 51–200, 201–500, 501–1000, >1000), and country of practice. Each surgeon performed two insertions, one left and one right, in mirror‐symmetric temporal bone models, with side order randomized. The models incorporated a completed mastoidectomy, a posterior tympanotomy, and a preformed bony implant bed, thereby focusing the procedure on the insertion workflow: seating the implant in the implant bed, advancing the electrode, sealing the round‐window niche, closing the posterior tympanotomy, and positioning the electrode lead within the mastoidectomy cavity. Surgeons were instructed to use their routine soft‐surgery technique. After each insertion, they rated perceived atraumaticity on a 1–10 scale.

A standard instrument set was available (SoftGrip forceps, regular and self‐closing, MED‐EL; angled surgical claw, MED‐EL; angled pick, Gyrus ACMI; standard forceps, Medicon and Bernstein; suction tube, Mediplast). Before starting, surgeons adjusted the microscope, seating, wrist supports, and instrument layout to their preferences. Porcine abdominal tissue was provided for packing at the round window and within the posterior tympanotomy. We used 20 original MED‐EL FLEX28 implants; each was inserted once for preconditioning and integrity checking, then reused for up to five insertions. Damaged arrays were replaced in two instances.

**FIGURE 1:**
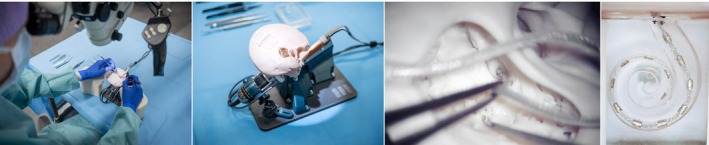
Experimental setup with right temporal bone model. Left to right: Complete setup in use; temporal bone model with hand rests removed; surgical microscope view; and recording of an inserted implant.

The temporal bone models reproduced the geometry obtained from human μCT data. Each model included a transparent, mechanically decoupled scala tympani lumen that was hydrophilically coated to reproduce realistic frictional behavior, yielding insertion forces in the range reported for cadaveric studies [8, 15]. Insertion force was measured with a uniaxial load cell aligned to the insertion axis (resolution, 0.1 mN; sampling, 80 Hz). Intracochlear pressure was measured apically (resolution, 0.1 Pa; sampling, 30 Hz). Electrode motion within the scala tympani was recorded by a digital microscope at 30 frames/s, and the surgical microscope view was captured in parallel. All signals and videos were time‐synchronized and processed in Python using SciPy [16]. Electrode contacts were first detected with a custom‐trained YOLOv8 (ultralytics) model, and contact trajectories were refined by optical‐flow tracking (OpenCV). From the surgical view, we manually annotated the boundaries of the insertion and post‐insertion phases. The experimental set‐up is shown in Figure 1.

To quantify mechanical stress across the workflow, we define six metrics (three during insertion and three during post‐insertion) that capture complementary micro‐ and macro‐mechanical effects (overview in Table 1). (i) *Pressure exposure (insertion)* and (ii) *pressure exposure (post‐insertion)* are the time integrals of squared intracochlear pressure over the respective phases. Pressure transients are dominated by low frequency kinematics, where air–fluid coupling is of order 1 [17], thus exposures are reported in dB re 20 μPa. (iii) *Force variation* is the sum of peak prominences for all force peaks with prominence >1 mN, identified using SciPy's “signal.find_peaks”; to prevent boundary effects from truncated peaks, signals were zero‐padded at segment boundaries before peak detection. Larger values indicate more frequent and/or larger stress cycles. (iv) *Directionality* is the ratio of net forward electrode contact displacement to total path length during insertion (1 denotes purely forward motion; lower values reflect retractions and transverse excursions). (v) *Post‐insertion motion* is the average total accumulated contact displacement after nominal placement, capturing movement during sealing and cable management. (vi) *Final position* is the absolute angular difference between achieved insertion depth and the anatomically optimal target for this model and implant (540°), with larger deviations indicating suboptimal cochlear coverage and tonotopic alignment.

**TABLE 1:** Overview of trauma‐related metrics, their physical basis, and clinical relevance across the insertion and post‐insertion phases.

Phase	Metric	Measurement target	Clinical relevance	Context
Insertion	Force variation	Fluctuations in applied insertion force	Repeated load cycles on cochlear structural scaffolds (e.g., spiral lamina, basilar membrane)	Macroscopic
	Directionality	Transverse bulging and retraction behavior	Spatial distribution of mechanical stress along the cochlear spiral	Mesoscopic
	Pressure exposure	Intracochlear pressure energy during insertion	Hydromechanical trauma to sensory epithelia (e.g., hair cell stereocilia)	Microscopic
Post‐insertion	Position	Deviation from target insertion depth	Suboptimal cochlear coverage and frequency‐place mismatch	Macroscopic
	Motion	Unintended implant displacement after placement	Compression and shear load on cochlear microstructures	Mesoscopic
	Pressure exposure	Intracochlear pressure energy during sealing and handling	Hydromechanical trauma to sensory epithelia	Microscopic

For comparability across heterogeneous units and distributions, each metric was normalized via its cumulative distribution. Approximately Gaussian metrics (insertion/post‐insertion pressure exposure; directionality) were modeled with normal distributions, and skewed metrics (force variation, final position, post‐insertion motion) with log‐normal distributions. We evaluated either the cumulative density function (CDF, for “higher‐is‐better” metrics) or the complementary CDF, 1−CDF (for “lower‐is‐better”), yielding scores in $\left[0,1\right]$ where 0.5 corresponds to the sample median and higher values indicate more favorable outcomes. The composite soft‐surgery score was the arithmetic mean of the six normalized metrics, providing a single, interpretable measure of mechanical stress from 0 (worst) to 1 (best).

Statistical analyses were performed in Python (SciPy). We used a significance threshold of p<0.05. Associations were assessed with Spearman's rank correlation (ρ); differences between distributions with the Kolmogorov–Smirnov test; and multi‐group comparisons with the Kruskal–Wallis H‐test, followed, when significant, by pairwise Kolmogorov–Smirnov tests. Variation was summarized by the quartile coefficient of dispersion, $\mathrm{QCD}=\frac{Q_{3}-Q_{1}}{Q_{3}+Q_{1}}$. For pressure‐based measures, QCD was computed in the linear pressure domain to respect the logarithmic nature of decibels. Maximal pressure instances were defined as the peak of the 1 s moving‐average–smoothed squared pressure signal.

### 2.1 Ethics Statement

This in vitro study involved no patients or patient data and was conducted exclusively in artificial temporal bone models. According to the Swiss Human Research Act, it does not fall within the scope of research requiring formal ethics committee approval. All participating surgeons provided informed consent for the anonymized analysis and publication of their performance data.

## 3 Results

All participants had clinical CI experience (median 20 years; interquartile range 8 years; range 0–40 years) and represented 11 countries; 74% had performed >200 CI surgeries and 27% >1000.

**FIGURE 2:**
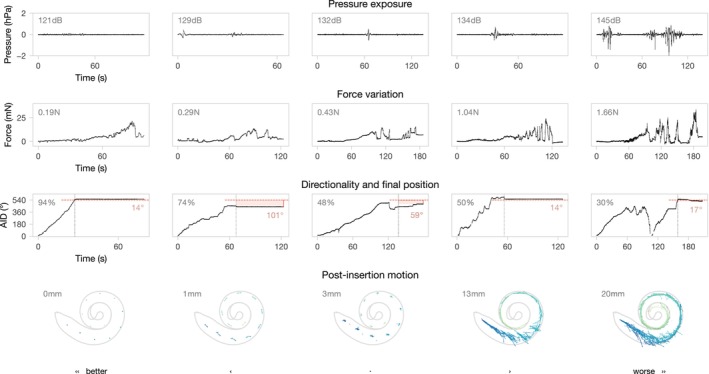
Illustration of the evaluated metrics, with five representative examples arranged from good (left) to bad (right). The top row shows recorded pressure, quantified by pressure exposure, that is, the cumulative pressure energy delivered into the cochlea. The second row depicts the applied insertion force, with force variation quantifying the number and strength of force peaks. The third row displays angular insertion depth over time, highlighting differences in directionality, where retractions lead to worse scores. Vertical bars denote the end of the insertion phase. Final position, defined as the deviation from the target insertion depth, is visualized in red (ordering in this row follows directionality and does not reflect the final position metric). The bottom row shows the post‐insertion motion, visualized as quiver plots. Inset quantities represent the respective metric values. Different procedures are shown across columns to illustrate representative cases for each metric.

We observed substantial variability across all quantitative metrics. Figure 2 illustrates force, pressure, insertion angle, and implant motion for representative cases. For force variation, final position, and post‐insertion motion, the interquartile range exceeded the respective median values. Pressure exposure also covered a wide dynamic range; the interquartile range exceeded 6 dB in both insertion and post‐insertion phases. We verified that high pressure exposure was not driven by integration of baseline pressure over time by computing a peak‐based pressure metric, which reproduced the same results. In addition, no learning effect was observed: the difference in soft‐surgery score between the first and second insertion was 0% ± 20%. Descriptive statistics for all metrics are summarized in Table 2.

**TABLE 2:** Descriptive statistics of the computed quantitative metrics.

Metric	Median	IQR	Range	Dispersion
Force variation (mN)	496	569	89.9–3297	0.51
Directionality (%)	65.6	28.0	19.8–94.9	0.24
Pressure exposure ins (dB)	131	6.64	109–145	0.64
Pressure exposure post (dB)	115	6.68	105–157	0.65
Final position (°)	48.1	61.5	1.59–219	0.57
Motion (mm)	1.21	2.28	0.040–20.0	0.67

Abbreviations: dispersion: Quartile coefficient of dispersion, IQR: Interquartile range.

### 3.1 Experience & Self‐Assessment

**FIGURE 3:**
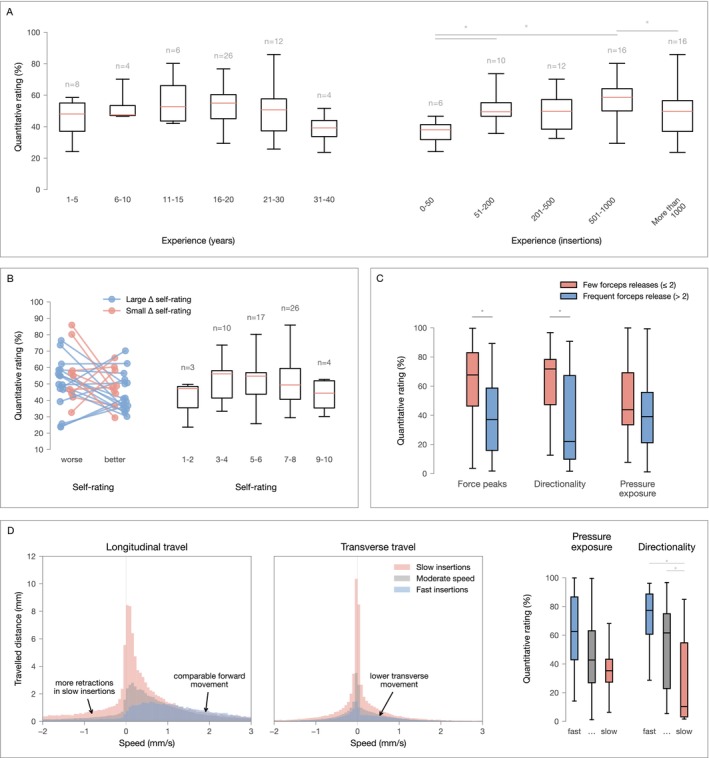
Quantitative analysis of surgeon performance and handling behaviors. All metrics are normalized from 0 to 1 (higher is better). (A) Soft‐surgery (quantitative trauma) score versus surgical experience in years (left) and lifetime cochlear implantations (right); (B) Self‐ratings versus quantitative soft‐surgery scores. (left), and within‐surgeon comparison of two insertions showing self‐rating differences versus differences in quantitative scores (right). (C) Force peak, pressure exposure, and directionality scores stratified by the number of forceps release events per insertion. (D) Longitudinal (left) and transverse (center) electrode speeds for slow, moderate, and fast insertions, and corresponding pressure exposure and directionality scores (right).

Surgeons with less than 50 lifetime insertions performed significantly worse than more experienced colleagues, whereas the 501–1000 group achieved the best composite soft‐surgery scores; performance in the >1000 group declined insignificantly. When experience was indexed by years of practice, the same trend was present but did not reach statistical significance. These results are presented in Figure 3A. Self‐assessment proved unreliable: self‐ratings did not correlate with any quantitative metric (ρ=−0.08, p=0.53), and procedures with the highest self‐ratings were frequently below average (subplot, Figure 3B, right). Moreover, surgeons were unable to identify which of their two insertions was quantitatively superior (ρ=0.07, p=0.60), even when their self‐rating difference was large (≥3 out of 10); paired differences are shown in the left panel of the same figure. Average self‐ratings differed across countries, indicating potential cultural effects on the interpretation of the numerical scale.

### 3.2 Surgical Handling

Maximal post‐insertion pressure and motion events were classified by handling task (coiling/sealing/packing) and, independently, by instrument maneuver (forceps release/electrode re‐grasp); classifications could overlap. For maximal‐pressure events, the handling task was most frequently coiling (38%), followed by sealing (22%) and packing (15%). In the overlapping maneuver coding, 15% of maximal‐pressure events coincided with forceps release and 5% with electrode grasping. For maximal‐motion events, the handling task was most frequently coiling (47%), followed by sealing (15%) and packing (13%). In the overlapping maneuver coding, 23% coincided with forceps release and 8% with electrode grasping.

Regarding insertion speed, between the fastest quartile (<75 s; median 52 s, IQR 15 s) and the slowest quartile (>200 s; median 233 s, IQR 168 s), net forward travel was similar, but slow insertions exhibited more frequent halts and retractions and substantially greater transverse motion. The second to third quartile group showed intermediate values across metrics. Directionality and pressure exposure were significantly worse in the slow group; distributions are shown in Figure 3D. Instrument handling showed a similar pattern. Frequent releasing and re‐grasping of the electrode worsened both force variation and directionality. Using a threshold of two releases yielded balanced groups (31 and 29 procedures; counts include the final release after the insertion): median force variation was 373 mN for ≤2 releases versus 684 mN for ≥3 releases, and median directionality was 71% versus 45%, respectively. Pressure exposure showed no significant difference between groups (medians 131 and 132 dB); see Figure 3C.

## 4 Discussion

Soft‐surgery principles are widely promoted and supported by a growing body of experimental and clinical work on specific techniques; however, objective quantification of phase‐specific mechanical stress using a unified set of metrics remains limited. Using a mechanically representative artificial temporal bone with multimodal sensing, we objectively quantified mechanical stress with six phase‐specific metrics that capture complementary trauma mechanisms. In doing so, we analyzed implantation behavior across 30 internationally recognized surgeons and linked concrete aspects of technique to measurable intracochlear stress.

Three observations emerge. *First*, even in a highly standardized environment, surgeon behavior produced wide dispersion in all metrics, underscoring that technique materially influences intracochlear pressure, force profiles, and electrode positioning. *Second*, surgeons were poor self‐assessors: subjective ratings did not track objective outcomes. *Third*, modifiable behaviors correlated with performance: excessively slow insertions and frequent regrasping were associated with greater mechanical stress, whereas steadier, more continuous advancement and fewer releases were favorable. Unlike prior single‐center work, we did not detect a consistent effect of post‐insertion step order in this larger cohort.

### 4.1 Learnability and Self‐Assessment

Our findings indicate that surgical handling plays a decisive role in cochlear implantation outcomes. Even under strictly standardized conditions, procedures varied considerably; most metrics showed wide dispersion, with the quartile coefficient of dispersion exceeding 0.5 for all but directionality. A volume–performance relationship emerged: surgeons with 0–50 lifetime insertions achieved significantly lower soft‐surgery scores, while scores were broadly similar among higher‐volume surgeons; the 501–1000 group performed best. Performance in the >1000 group declined significantly, but weakly. Performance difference versus years of practice was not statistically significant, suggesting that continuous hands‐on exposure has greater impact than time in practice alone.

Further, our data uncovered that self‐assessment is unreliable. Surgeons' subjective ratings showed no meaningful correlation with objective performance metrics, and they were unable to determine which of their two insertions achieved superior quantitative outcomes. This limitation is likely attributable to the sub‐perceptual nature of the relevant mechanical events: force peaks often fall below the threshold of human tactile perception [18], and the micrometer‐scale electrode micromotions that generate pressure transients are imperceptible during surgery. These findings underscore the need for surgical guidance based on objective, quantifiable feedback rather than subjective judgment. Promising solutions include real‐time electrocochleography (ECochG) and impedance‐based monitoring, which have demonstrated potential for intraoperative application [19, 20, 21, 22].

### 4.2 Modifiable Surgical Aspects

#### 4.2.1 Insertion Speed

Current guidelines favor “slow” advancement but lack operational thresholds. Consistent with prior observations [23], most surgeons used slow speeds (median duration 112 s ≈15 mm/min). Nevertheless, our data indicate that *excessively* slow insertions are counterproductive: compared with the fastest quartile (<75 s, ≈22 mm/min), prolonged insertions (>200 s) showed more halts and retractions, worse directionality, and higher pressure exposure. Kesler et al. reported that truly continuous forward motion is not achievable below ∼52 mm/min [24], which helps explain the observed start–stop behavior at slow speeds. These findings support steady, uninterrupted advancement within a slow‐to‐moderate range, rather than deliberate ultra‐slow motion. Importantly, they do not imply a return to high speeds characteristic of earlier eras [25], which were not represented here.

#### 4.2.2 Forceps Handling

Releasing and regrasping the array increased force variation and reduced directionality, consistent with elastic recoil and unintended back‐out during grasp transitions (see also [10]). Planning the grip and trajectory to minimize releases is a concrete, teachable behavior with measurable benefit.

#### 4.2.3 Post‐Insertion Phase

Our findings indicate that clinically relevant mechanical stress and implant migration can arise after nominal placement. These observations are consistent with previous studies in the same setup [26] and recent force‐based measurements by Cramer et al. [27], who demonstrated pronounced insertion force peaks during post‐insertion handling, particularly during electrode release and lead fixation. Although earlier work suggested sealing the facial recess before cable coiling [10], we did not reproduce a consistent benefit, likely because the stabilizing effect depends on firm tissue packing, which varied across surgeons. These results motivate explicit attention to post‐insertion handling in protocols and tool design. This becomes especially relevant with the use of motorized insertion tools, which only address the insertion phase [26, 27].

#### 4.2.4 Implications for Soft‐Surgery and Training

Traditional recommendations emphasize slowness and gentleness but lack quantitative definitions, and surgeons cannot reliably gauge performance intraoperatively. Our findings argue for evidence‐based targets (e.g., acceptable ranges for directionality, force variation, and pressure exposure) and for training paradigms that provide immediate, quantitative feedback. Artificial models such as the one used here enable objective assessment and iterative practice. In the operating theater, real‐time ECochG and impedance telemetry can extend this principle to patient care [20, 28]. Together, these approaches can refine soft‐surgery from qualitative advice to measurable standards, focusing on steady advancement, minimal regrasping, and careful post‐insertion handling to minimize intracochlear stress.

## 5 Limitations

This study was conducted using a single artificial cochlear anatomy, one implant type, and one electrode array design. While this approach maximized comparability across participants, it does not reflect the full anatomical variability encountered in clinical practice or the diverse mechanical behaviors of different electrode designs. Each surgeon performed only two insertions, which limited our ability to assess intra‐operator variability or detect learning effects within the experiment. Although we used an established artificial model that reproduces many relevant aspects of cochlear geometry and frictional conditions, it does not capture intracochlear microanatomy or tissue‐specific viscoelastic properties.

Moreover, the defined metrics and the combined soft‐surgery score represent an effort to reduce complex implantation dynamics to a limited set of quantitative descriptors. This enables objective comparison without implying a level of mechanistic specificity that our experimental setup cannot support. In particular, different trauma mechanisms may result in similar metric values: for example, a brief, localized force peak with the potential to disrupt cochlear structures may yield comparable scores to more distributed loading along the electrode array, which may instead induce trauma through sustained compression of the cochlear microvasculature. Within these constraints, the selected metrics provide a pragmatic and internally balanced representation of mechanically relevant behavior, which we consider appropriate for the present experimental setup and for comparative analysis across surgeons and techniques.

Finally, the study was observational in nature, allowing surgeons to operate mostly according to their preferred technique. Although this provided a realistic representation of individual practice styles, it precluded systematic testing of specific surgical variables. Furthermore, some surgeons may routinely use alternative fixation techniques (e.g., clip, suture, drilled slit) that were not available in our setup, which may have influenced their performance. Future work should therefore adopt a more controlled design, in which individual factors such as insertion speed, forceps handling, and post‐insertion steps are systematically varied. Such studies would allow for clearer causal inference and provide more granular evidence to guide refinement of soft‐surgery protocols.

## 6 Conclusion

This study demonstrates that surgical behavior is a decisive determinant of intracochlear mechanical stress in cochlear implantation. Excessively slow insertions, frequent regrasping, and uncontrolled post‐insertion motion were associated with higher pressure, force, and directionality penalties, whereas steady advancement and minimal regrasping yielded more favorable outcomes. Because surgeons cannot reliably perceive these microscopic events, structured training is essential: (i) key surgical aspects that protect cochlear integrity must be explicitly taught, (ii) their execution should be practiced and quantified in a preclinical model environment that provides immediate feedback, and (iii) translation into the operating theater should be supported by real‐time monitoring of cochlear function. Such an evidence‐based, feedback‐driven approach will enable measurable improvements in technique, help preserve residual hearing, and optimize cochlear implant outcomes.

## Funding

The authors have nothing to report.

## Conflicts of Interest

The authors declare no conflicts of interest.

## Data Availability

The data that support the findings of this study are available on request from the corresponding author. The data are not publicly available due to privacy or ethical restrictions.

## References

[1] E. Lehnhardt , “Intracochlear Placement of Cochlear Implant Electrodes in Soft Surgery Technique,” HNO 41, no. 7 (1993): 356–359. 8376183

[2] R. Laszig , G. J. Ridder , and M. Fradis , “Intracochlear Insertion of Electrodes Using Hyaluronic Acid in Cochlear Implant Surgery,” Journal of Laryngology & Otology 116, no. 5 (2002): 371–372, 10.1258/0022215021910816. 12080996

[3] T. Lenarz and K. Dalkowski , Cochlear Implantation: The Hannover Guideline (Endo‐Press, 2006).

[4] D. R. Friedland and C. Runge‐Samuelson , “Soft Cochlear Implantation: Rationale for the Surgical Approach,” Trends in Amplification 13, no. 2 (2009): 124–138, 10.1177/1084713809336422. 19447766 PMC4111526

[5] N. T. Greene , J. K. Mattingly , R. M. Banakis Hartl , D. J. Tollin , and S. P. Cass , “Intracochlear Pressure Transients During Cochlear Implant Electrode Insertion,” Otology & Neurotology 37, no. 10 (2016): 1541–1548, 10.1097/MAO.0000000000001232. 27753703 PMC5104176

[6] T. Ishii , M. Takayama , and Y. Takahashi , “Mechanical Properties of Human Round Window, Basilar and Reissner's Membranes,” Acta Oto‐Laryngologica 115, no. sup519 (1995): 78–82, 10.3109/00016489509121875. 7610897

[7] P. Aebischer , G. Mantokoudis , S. Weder , L. Anschuetz , M. Caversaccio , and W. Wimmer , “In‐Vitro Study of Speed and Alignment Angle in Cochlear Implant Electrode Array Insertions,” IEEE Transactions on Biomedical Engineering 69, no. 1 (2022): 129–137, 10.1109/TBME.2021.3088232. 34110987

[8] P. Aebischer , S. Weder , G. Mantokoudis , M. Vischer , M. Caversaccio , and W. Wimmer , “A Sleeve‐Based, Micromotion Avoiding, Retractable and Tear‐Opening (SMART) Insertion Tool for Cochlear Implantation,” IEEE Transactions on Biomedical Engineering 70, no. 3 (2023): 860–866, 10.1109/TBME.2022.3204069. 36063524

[9] C. G. Wright and P. S. Roland , “Temporal Bone Microdissection for Anatomic Study of Cochlear Implant Electrodes,” Cochlear Implants International 6, no. 4 (2005): 159–168, 10.1179/cim.2005.6.4.159. 18792334

[10] P. Aebischer , S. Weder , M. Vischer , G. Mantokoudis , M. Caversaccio , and W. Wimmer , “Uncovering Vulnerable Phases in Cochlear Implant Electrode Array Insertion: Insights From an In Vitro Model,” Otology & Neurotology 45, no. 4 (2024): e271–e280, 10.1097/MAO.0000000000004130. 38346807

[11] S. Weder , C. Bester , A. Collins , C. Shaul , R. J. Briggs , and S. O'Leary , “Toward a Better Understanding of Electrocochleography: Analysis of Real‐Time Recordings,” Ear and Hearing 41, no. 6 (2020): 1560–1567, 10.1097/AUD.0000000000000871. 33136631

[12] R. M. Knoll , D. R. Trakimas , M. J. Wu , et al., “Intracochlear New Fibro‐Ossification and Neuronal Degeneration Following Cochlear Implant Electrode Translocation: Long‐Term Histopathological Findings in Humans,” Otology & Neurotology 43, no. 2 (2022): e153–e164, 10.1097/MAO.0000000000003402. 35015749

[13] C. G. Wright and P. S. Roland , Cochlear Anatomy via Microdissection With Clinical Implications (Springer International Publishing, 2018), 10.1007/978-3-319-71222-2.

[14] T. Weller , M. E. Timm , T. Lenarz , and A. Büchner , “Cochlear Coverage With Lateral Wall Cochlear Implant Electrode Arrays Affects Post‐Operative Speech Recognition,” PLoS One 18, no. 7 (2023): e0287450, 10.1371/journal.pone.0287450. 37437046 PMC10337941

[15] P. Aebischer , M. Caversaccio , and W. Wimmer , “Fabrication of Patient‐Specific Human Anatomy‐Based Scala Tympani Models With a Hydrophilic Coating for Cochlear Implant Insertion Experiments,” Hearing Research 404 (2021): 8, 10.1016/j.heares.2021.108205. 33618163

[16] P. Virtanen , R. Gommers , T. E. Oliphant , et al., “SciPy 1.0: Fundamental Algorithms for Scientific Computing in Python,” Nature Methods 17 (2020): 261–272, 10.1038/s41592-019-0686-2. 32015543 PMC7056644

[17] H. H. Nakajima , W. Dong , E. S. Olson , S. N. Merchant , M. E. Ravicz , and J. J. Rosowski , “Differential Intracochlear Sound Pressure Measurements in Normal Human Temporal Bones,” Journal of the Association for Research in Otolaryngology 10, no. 1 (2009): 23–36, 10.1007/s10162-008-0150-y. 19067078 PMC2644388

[18] L. B. Kratchman , D. Schuster , M. S. Dietrich , and R. F. Labadie , “Force Perception Thresholds in Cochlear Implantation Surgery,” Audiology and Neurotology 21, no. 4 (2016): 244–249, 10.1159/000445736. 27576674 PMC5508863

[19] R. R. Andonie , W. Wimmer , R. A. Wildhaber , M. Caversaccio , and S. Weder , “Real‐Time Feature Extraction From Electrocochleography With Impedance Measurements During Cochlear Implantation Using Linear State‐Space Models,” IEEE Transactions on Biomedical Engineering 70, no. 11 (2023): 3137–3146, 10.1109/TBME.2023.3276993. 37195836

[20] R. R. Andonie , W. Wimmer , S. Schraivogel , G. Mantokoudis , M. Caversaccio , and S. Weder , “Electrocochleography in Cochlear Implant Recipients: Correlating Maximum Response With Residual Hearing,” Ear and Hearing 46, no. 1 (2025): 16–23, 10.1097/aud.0000000000001546. 39010266 PMC11637568

[21] R. R. Andonie , W. Wimmer , R. A. Wildhaber , G. Mantokoudis , M. Caversaccio , and S. Weder , “Electrocochleography Latency: Correlation With Electrode Position During Cochlear Implantation,” Ear and Hearing 46, no. 5 (2025): 1130–1140, 10.1097/AUD.0000000000001652. 40001274 PMC12352560

[22] R. Andonie , M. Caversaccio , S. Weder , and W. Wimmer , “Impedance‐Based Tissue Response Modeling for the Prediction of Hearing Preservation After Cochlear Implantation,” Computers in Biology and Medicine 196 (2025): 110626, 10.1016/j.compbiomed.2025.110626. 40614514

[23] G. P. Rajan , G. Kontorinis , and J. Kuthubutheen , “The Effects of Insertion Speed on Inner Ear Function During Cochlear Implantation: A Comparison Study,” Audiology and Neurotology 18, no. 1 (2013): 17–22, 10.1159/000342821. 23006502

[24] K. Kesler , N. P. Dillon , L. Fichera , and R. F. Labadie , “Human Kinematics of Cochlear Implant Surgery: An Investigation of Insertion Micro‐Motions and Speed Limitations,” Otolaryngology‐Head and Neck Surgery 157, no. 3 (2017): 493–498, 10.1177/0194599817704391. 28508720

[25] G. Kontorinis , T. Lenarz , T. Stöver , and G. Paasche , “Impact of the Insertion Speed of Cochlear Implant Electrodes on the Insertion Forces,” Otology & Neurotology 32, no. 4 (2011): 565–570, 10.1097/MAO.0b013e318219f6ac. 21478788

[26] P. Aebischer , L. Anschuetz , M. Caversaccio , G. Mantokoudis , and S. Weder , “Quantitative In‐Vitro Assessment of a Novel Robot‐Assisted System for Cochlear Implant Electrode Insertion,” International Journal of Computer Assisted Radiology and Surgery 20, no. 2 (2024): 323–332, 10.1007/s11548-024-03276-y. 39352456 PMC11807918

[27] J. Cramer , G. Böttcher‐Rebmann , M. Fröhlich , et al., “Robot‐Assisted Versus Manual: Intracochlear Forces During and After Cochlear Implant Electrode Insertion Show Benefits of Automation and Electrode Guidance,” Otology & Neurotology 47, no. 1 (2026): 81–89, 10.1097/MAO.0000000000004636. 41054951

[28] P. Aebischer , S. Meyer , M. Caversaccio , and W. Wimmer , “Intraoperative Impedance‐Based Estimation of Cochlear Implant Electrode Array Insertion Depth,” IEEE Transactions on Biomedical Engineering 68, no. 2 (2021): 545–555, 10.1109/TBME.2020.3006934. 32746052

